# Post Endoscopic Retrograde Cholangiography Bilateral Loss of vision: A Case Report

**DOI:** 10.31729/jnma.6548

**Published:** 2021-10-31

**Authors:** Ashis Pun, Kamlesh Kumar Sah, Sunil Kumar Sah, Ramjee Bastola

**Affiliations:** 1 Department of Surgery, Bharatpur Hospital, Chitwan, Nepal; 2 Department of Medicine, Bharatpur Hospital, Chitwan, Nepal; 3 Department of Psychiatry, Bharatpur Hospital, Chitwan, Nepal

**Keywords:** *endoscopic retrograde cholangiopancreatography*, *lateral geniculate body*, *visual acuity*

## Abstract

Endoscopic retrograde cholangiopancreatography is an invasive endoscopic procedure done more often for therapeutic rather than diagnostic purposes. There are various complications of this procedure like pancreatitis, cholangitis, hemorrhage, perforation and other rare adverse events. In this case report, we discuss a case of a 40 years female who was referred to our center for endoscopic retrograde cholangiography. After the procedure she complained of bilateral loss of vision which was an unknown complication to us. But after looking back to literature we found two such case reports attributed to isolated bilateral lateral geniculate body infarct.

## INTRODUCTION

Endoscopic Retrograde Cholangiopancreatography (ERCP) is one of the frequently used treatment modality for various pancreatobiliary problems whose indication can be categorized into stone diseases, ampullary abnormalities and biliary/pancreatic duct abnormalities. Among these, choledocholithiasis holds the most common indication of ERCP. Choledocholithiasis is seen in about 15% of patient with cholelithiasis.^1^

Frequent complications following ERCP includes-pancreatitis (3% to 10%), cholangitis (0.5% to 3%), hemorrhage (0.3% to 2%) and perforation (0.08% to 0.6%); however, there are other rare incidents which includes, approximately 1 % like cardiopulmonary, air embolism, ileus, intrahepatic sub capsular hematoma, hepatic abscess, splenic hematoma, pseudocyst infection, biliary or pancreatic duct fistula and reaction to contrast.^2^

## CASE REPORT

A 40 years female was referred to our centre with diagnosis of cholelithiasis and choledocholithiasis for ERCP, and was admitted to Surgery Department, Bharatpur Hospital. She came with a complaint of abdominal pain for one month, which was localized in the epigastric region; intermittent and colicky in nature, accompanied by nausea but had no vomiting. She had no change in bowel habits. Patient was otherwise well with no significant medical and surgical history. She had no icterus and her vitals were stable. On per abdominal examination, minimal tenderness was present in the epigastric region. Her routine investigations haemoglobin 12 gm/dl, platelet 145000/cumm, random blood sugar 107mg/dl, serum creatinine 0.5 mg/dl, alanine aminotransferase (ALT) 40 IU/L, aspartate aminotransaminase (AST) 45 IU/L were within normal limit. Ultrasonography showed cholelithiasis with solitary choledocholithiasis, common bile duct diameter (9mm). Magnetic Retrograde Cholangiopancreatography (MRCP) revealed: Cholelithiasis with at least 3 mid CBD stones, CBD-9mm.

**Figure 1:**
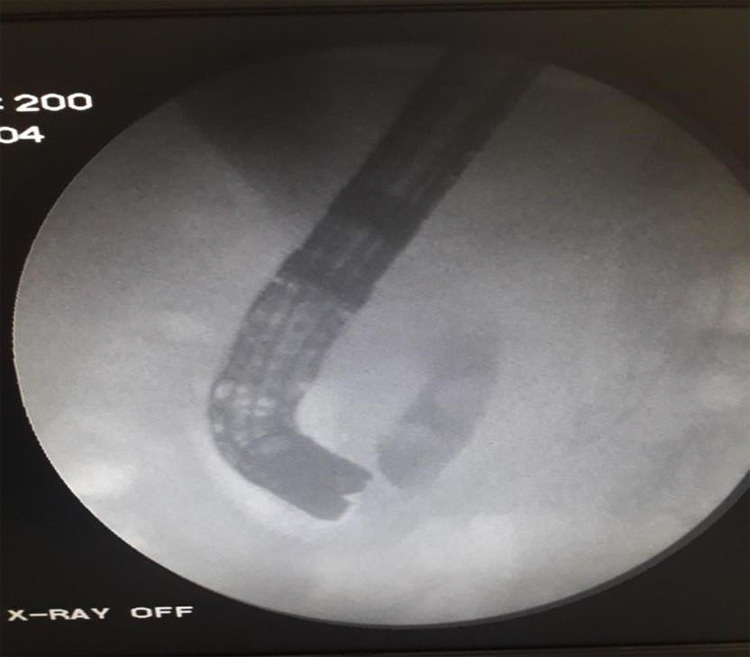
Showing filling defect in Cholangiogram.

Under the diagnosis of cholelithiasis with choledocholithiasis, she was planned for ERCP and stone extraction. During ERCP needle knife sphincterotomy had to be done to facilitate CBD cannulation. Stones were extracted with subsequent Balloon sweep (Figure 1).

**Figure 2:**
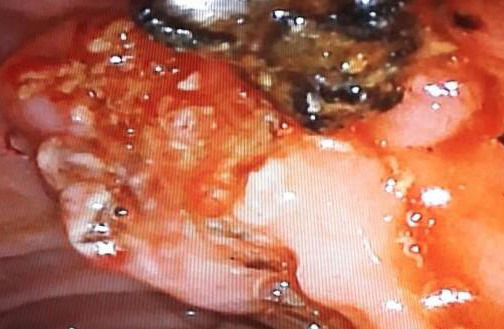
Showing stone extracted from ampulla after sphincterotomy stones.

No filling defect in check cholangiogram (Figure 2).

We routinely give 75 mg diclofenac during the procedure to reduce pain.

On the 1^st^ postoperative day the patient complained of gradual painless bilateral loss of vision. She had no complaint of abdominal pain. He r neurological assessment was normal except the complaint of bilateral visual loss. Patient's serum amylase and full blood count was sent which came within normal range. Then, on 2nd postoperative day, she was sent to the Ophthalmology department. Ophthalmology reported normal fundoscopic findings. After this, the patient was scheduled for Magnetic Resonance Imaging (MRI) head. However, on the next day, the patient claimed to have regained her bilateral vision and refused an MRI as she had a history of claustrophobic attack during MRCP. She was discharged on 4th postoperative day and was referred back to the respective centre for cholecystectomy. She was called for follow-up after 1 month but she came after 1 year. She had not done cholecystectomy and during this follow up, we did her laparoscopic cholecystectomy and the postoperative period was uneventful.

## DISCUSSION

Visual loss following endoscopic retrograde cholangiopancreatography (ERCP) is a rare complication with very few case reports in literature. The possible explanation of bilateral visual loss following ERCP includes isolated bilateral lateral geniculate body (LGB) infraction, acute pancreatitis with Purtscher's retinopathy, drugs, hysteria and contrast reaction.

In 2006, Ophthalmologist Baker CF, et al. reported post ERCP vision loss in a 29 years female who had undergone ERCP with diagnosis of choledocholithiasis. Vision loss was found to be due to isolated bilateral LGB infarct which was detected in the MRI head. Besides, the author had also mentioned an episode of procedural hypotension which may be a cause of vision loss. The patient's vision was improving during 13 days; however, her long term follow up is not mentioned. In this case, patient's migraine with typical aura can be an aggravating factor as migraine in middle aged women are prone to ischemic stroke commonly bilateral ischemic posterior cerebral artery strokes. Migraine's strokes are thought to cause vasospasm, which may hinder blood flow, promote thrombosis and furthermore, activating platelets and triggering endothelial cells to release thrombosis promoting factors.^3^

Similarly, in 2016, Gastroenterologist Bartel MJ, et al. also reported a visual loss in 22 years female following ERCP due to isolated bilateral LGB infarct. She had associated cholecystitis and developed pancreatitis after ERCP. He had also quoted a case report, by Mudumbai in 2007, of an 18-year-old female with pancreatitis and microangiopathy. She had only a medical history of oral contraceptive use and was intubated for severe pancreatitis. She was eventually extubated 8 days later when she reported bilateral blurred vision with visual field defect without any other neurological deficit. After 32 months, her visual acuity returned back to near normal with only slight visual field loss.

Subsequently authors have reported bilateral LBG damage due to syphilitic arteritis, LGB necrosis due to methanol toxicity and microangiopathic hemolytic anemia, bilateral geniculate myelinolysis due to rapid overcorrection of chronic hyponatremia, and bilateral aseptic geniculitis associated with severe diarrhoea.^4^

Purtscher's retinopathy is another rare condition that is associated with complement- activating systemic diseases such as acute pancreatitis which can lead to loss of vision. The possible pathophysiology thought to be coagulation and leuko embolization of retinal precapillary arterioles.^5^ Likewise, contrast can also cause blindness due to transient cortical blindness and hypotension. The putative mechanism of transient cortical blindness is disruption of the blood brain barrier, direct neuronal injury and vasoconstriction. However, vision lost have been found to resolve entirely within hours to 5 days.^6,7^

Even though, when we searched articles on post ERCP vision loss we only found 2 case reports and, in both cases, blindness was attributed to bilateral LGB damage, it was the least possible cause in our case because other reported cases of LGB damage took longer duration for visual acuity to come near normal even up to 32 months. On the contrary, our patient recovered suddenly within 3 days. The MRI brain of the patient would have explained better but our patient did not give consent for the MRI. Likewise, we also exclude Purtscher's retinopathy as the patient never had pancreatitis before and after the procedure. Although contrast reaction can be a possible cause in our case as the blindness was transient but, contrast induced vision loss were seen only in those cases where it was given intravenously. In ERCP, contrast is given in the pancreaticobiliary system and it is least likely to enter the vascular system. Whereas, in drugs, unlike other non-steroidal anti-inflammatory drugs only indomethacin has been found to affect LGB. In our case we never used Indomethacin only Diclofenac was given intramuscularly.

Hence, the only possible explanation for bilateral vision loss in our case was found to be dissociative neurological symptom disorder with visual disturbance; traditionally well known as hysterical blindness. This disorder is coded as 6B60.0 in ICD 11, the international classification of diseases for morbidity and mortality statistics. The visual disturbance (blindness here), is not explainable by any nervous system or other medical or mental behavioral issues.^8^

ERCP is an invasive procedure so the endoscopist must be aware of its various complications. Bilateral vision loss can also occur as a squeal of ERCP.
